# Workplace Violence in Healthcare: Patterns, Prevalence, and Psychological Effects

**DOI:** 10.1192/j.eurpsy.2026.11244

**Published:** 2026-07-31

**Authors:** A. Aloui, I. Kacem, N. Ganoun, A. chouchane, M. Bouhoula, R. Ghammem, M. Makhloufi, O. El Maalel, S. Chatti, H. Kalboussi

**Affiliations:** 1 Department of Occupational Medicine; 2 Epidemiological department, Farhat Hached University Hospital , Sousse, Tunisia

## Abstract

**Introduction:**

Workplace violence in healthcare settings is an increasing problem affecting the safety and well-being of healthcare staff. It can manifest as physical, verbal, or psychological abuse, leading to stress, burnout, and reduced quality of care.

**Objectives:**

To determine the prevalence of workplace violence in hospital settings and its different forms and psychological effects

**Methods:**

This was an analytical cross-sectional study conducted among healthcare professionals at Farhat Hached University Hospital. Data were collected using a pre-established form covering participants’ socio-professional characteristics and the forms of violence experienced and its psychological effects.

**Results:**

Among the 500 healthcare workers approached during the study period, 362 participated, representing a response rate of 72.4%. The mean age was 37.3 ± 8.71 years, with a predominance of females (59.4%). Paramedical staff constituted the majority (54.4%), with a median professional experience of 7 years. In our study population, 235 participants (64.9%) experienced external violence (from non-hospital personnel), with verbal abuse being the most frequent form (54.4%). Additionally, 256 hospital staff (70.7%) experienced internal violence (from colleagues), predominantly verbal (94.5%). Most participants (78.2%) witnessed internal or external violence, mainly verbal in nature (76%). Among respondents, 312 (86.2%) reported physical or psychological symptoms during the past 12 months, with fatigue (58.6%) and nervousness (52.5%) being the most common.

**Conclusions:**

Workplace violence in healthcare is frequent and negatively affects the physical and psychological health of staff. Understanding its prevalence and manifestations is crucial to developing effective prevention strategies.

**Disclosure of Interest:**

None Declared

